# Comparative flavonoid profile of orange (*Citrus sinensis*) flavedo and albedo extracted by conventional and emerging techniques using UPLC-IMS-MS, chemometrics and antioxidant effects

**DOI:** 10.3389/fnut.2023.1158473

**Published:** 2023-06-06

**Authors:** Sherif M. Afifi, Recep Gök, Ingo Eikenberg, Dennis Krygier, Eric Rottmann, Anne-Sophie Stübler, Kemal Aganovic, Silke Hillebrand, Tuba Esatbeyoglu

**Affiliations:** ^1^Institute of Food Science and Human Nutrition, Gottfried Wilhelm Leibniz Universität Hannover, Hannover, Germany; ^2^Pharmacognosy Department, Faculty of Pharmacy, University of Sadat City, Sadat City, Egypt; ^3^Institute of Food Chemistry, Technische Universität Braunschweig, Braunschweig, Germany; ^4^Symrise AG, Holzminden, Germany; ^5^German Institute of Food Technologies (DIL e.V.), Quakenbrück, Germany

**Keywords:** albedo, flavedo, extraction, metabolomics, ultrasonic, high pressure processing, pulsed electric field, polymethoxy flavones

## Abstract

**Introduction:**

*Citrus* fruits are one of the most frequently counterfeited processed products in the world. In the juice production alone, the peels, divided into flavedo and albedo, are the main waste product. The extracts of this by-product are enriched with many bioactive substances. Newer extraction techniques generally have milder extraction conditions with simultaneous improvement of the extraction process.

**Methods:**

This study presents a combinatorial approach utilizing data-independent acquisition-based ion mobility spectrometry coupled to tandem mass spectrometry. Integrating orthogonal collision cross section (CCS) data matching simultaneously improves the confidence in metabolite identification in flavedo and albedo tissues from *Citrus sinensis*. Furthermore, four different extraction approaches [conventional, ultrasonic, High Hydrostatic Pressure (HHP) and Pulsed Electric Field (PEF)] with various optimized processing conditions were compared in terms of antioxidant effects and flavonoid profile particularly polymethoxy flavones (PMFs).

**Results:**

A total number of 57 metabolites were identified, 15 of which were present in both flavedo and albedo, forming a good qualitative overlapping of distributed flavonoids. For flavedo samples, the antioxidant activity was higher for PEF and HHP treated samples compared to other extraction methods. However, ethyl acetate extract exhibited the highest antioxidant effects in albedo samples attributed to different qualitative composition content rather than various quantities of same metabolites. The optimum processing conditions for albedo extraction using HHP and PEF were 200 MPa and 15 kJ/kg at 10 kV, respectively. While, HHP at medium pressure (400 MPa) and PEF at 15 kJ/kg/3 kV were the optimum conditions for flavedo extraction.

**Conclusion:**

Chemometric analysis of the dataset indicated that orange flavedo can be a valid source of soluble phenolic compounds especially PMFs. In order to achieve cross-application of production, future study should concentrate on how citrus PMFs correlate with biological engineering techniques such as breeding, genetic engineering, and fermentation engineering.

## 1. Introduction

Having sweet taste and aroma (1), *Citrus* fruits, members of the Rutaceae family, rank fourth among the most widely consumed fruits in the world after apples, bananas, and watermelons (2). Worldwide, the production of oranges (*Citrus sinensis*) is steadily increasing. In 2021, the global production of oranges was about 49.3 Mio metric tons. Oranges are cultivated primarily in Brazil followed by China, the United States and Mexico, with world annual production of 39, 24, 8, and 6%, respectively (3). Commercial all year-round availability of oranges is supported through import (4). They are mostly consumed fresh, whereas only 4% of oranges are processed into juice. During consumption and processing of oranges, the non-edible compartments, especially the peel, are generated as a by-product. In the juice production alone, the peels, which can be divided into flavedo and albedo, are the main side stream products. They contains many bioactive substances like flavonoids (5), pectins (6), carotenoids (7), essential oils (8), and sugars (9), which are potential ingredients for food, pharmaceutical, cosmetic, and other industries. Previous recycling measures foresee the utilization of the dried organic mass as animal feed (10). Other approaches are aimed at extracting attractive ingredients, such as pectin, D-limonene and converting the soluble and insoluble sugars into bioethanol (9).

Main flavonoids in citrus fruits are flavones, flavanones and their polymethoxy derivatives, with a distinction being made between glycosidated and aglycones (11). Polymethoxy flavones (PMFs) have at least two methoxy groups on the flavonoid skeleton consisting of a benzene ring linked at position 2 of a benzopyran ring and may also contain hydroxyl groups or sugar moieties (5). The bioactive characteristics of PMFs, such as their anti-inflammatory (12), anti-proliferative (13), anti-obesity (14), anti-cancer (15), anti-diabetes (14), anti-fungal (16), anti-microbial (17), anti-viral (18), and neuro-protection (19) have led to their unique emergence in the *Citrus* genus (20).

The most commonly used solvent extraction technique is characterized by its simplicity and the possibility of using different solvents to achieve selectivity in advance. The disadvantage is the use of organic solvents under consideration of environmental protection and sustainability, as well as long extraction times. For these reasons, new extraction approaches and supportive technologies are suggested and investigated, such as ultrasound, pulsed electric fields and high hydrostatic pressure (21). Ultrasonic extraction (UAE), has already been investigated for extraction of bioactive compounds in apple pomace (22), strawberries (23), grapes (24), green tea leaves (25), and citrus peels (26). The major process parameters relevant for the UAE process are sonication temperature, time, and power (27). Modern extraction techniques generally aim for milder and more efficient extraction conditions with simultaneous improvement of the extraction process. Pulsed Electric Field (PEF) is another emerging technology described as a potential approach for improving mass transfer and extraction methods (28). In this technology, the electroporation causes rupture in the cell membranes through the applications of microseconds electrical pulses in an externally applied electric field. Depending on the process intensity, reversible or irreversible membrane disruption can be achieved (29). In addition to PEF, High Hydrostatic Pressure (HHP) represents an emerging treatment for food products with potential for retaining freshness and extending shelf-life. For this purpose, the food product is placed in its final packaging in the water-filled pressure chamber and subjected to the desired pressure (typically—up to technically possible limit of 600 MPa) for a few minutes (30). Because of the very high pressure, cell damages occur, which in turn can result in improved extraction performances. Both HHP and PEF have already been used in extraction procedures for grapes (31), moringa (32), red cherries (33), tea leaves (34), ginger (34), and tomatoes (35). The improvement of citrus juice quality was the focus of earlier investigations on the effects of the PEF or HHP procedure (36–38), whereas the citrus peel was sparingly handled and evaluated to optimize the aforementioned extraction techniques (39).

In recent years, ESI-IMS-QToF-MS^E^ in positive and negative modes has been established for characterization, identification, and quantifications in untargeted or targeted approach of different citrus species (5, 40, 41). Despite very good results, even the high-resolution chromatography and mass spectrometry reach their limits for very complex matrices and the large number of compounds. Especially isomeric and isobaric compounds cannot be separated by conventional LC–MS methods (42). Due to its high sensitivity, and rapid response, ion mobility spectrometry (IMS) has often been regarded as an analytical instrument offering an additional separation dimension for the measurement of isobaric and isomeric molecules when combined with liquid chromatography. Thus, various metabolites can be more easily detected and identified from chemical background, owing to the improvement in resolving power based on the size to charge ratio. Moreover, the measured CCS (collision cross section) value provides depth and detail, particularly when it is not associated to *m/z* because it is dependent on the molecular makeup of the metabolite and aids in the clear identification of the substances under study (43).

In order to illustrate the benefits and possible scalability of these so-called “enabling technologies,” the outcomes and efficacy of those technologies, such as UAE, PEE and HHP, are compared herein to both conventional technologies and between the new technologies themselves. To the best of our knowledge, no systematical published data exist on CCS values of PMFs. Therefore, it is anticipated that this study is the first attempt to use IMS-related parameter as a new dimension for revealing chemical composition of polymethoxy flavones of flavedo and albedo parts extracted by conventional and novel extraction techniques, i.e., PEF and HHP in the context of chemometrics and antioxidant effects.

## 2. Materials and methods

### 2.1. Chemicals

Methanol (p.a., min 99%), acetic acid (p.a., min. 99.0%), sodium acetate (anhydrous, p.a., min. 99.0%) and iron (III) chloride hexahydrate (p.a., min. 99.0%) were purchase from ChemSolute^®^ (Renningen, Germany). Ethyl acetate (≥99.7%, PESTINORM^®^), methanol (≥99.9%, HiPerSolv CHROMANORM^®^, super gradient), acetonitril (HiPerSolv CHROMANORM^®^ Reag, ≥99.95%, super gradient grade) were ordered from VWR International (Darmstadt, Germany). (±)-6-Hydroxy-2,5,7,8-tetramethylchromane-2-carboxylic acid (Trolox, 97.0%), 2,4,6-tris-(2-pyridyl)-s-triazin (TPTZ, ≥98%), sodium phosphate monobasic (p.a., anhydrous, ≥99.0%), 2,2-diphenyl-1-picrylhydrazyl (DPPH, 95%), standard flavonoids, and 2,2′-azobis-(2-methyl-propionamidine) dihydrochloride (AAPH, 97%), were purchased from Sigma-Aldrich (Schnelldorf, Germany). Furthermore, L-(+)-ascorbic acid (≥99%, p.a.), fluorescein disodium salt (C.I. 45,350) and sodium carbonate (≥99.5% anhydrous) were obtained from Carl Roth (Karlsruhe, Germany). Ethanol (absolute, min. 99.8%) was purchased from Walter-CMP (Kiel, Germany), sodium phosphate dibasic dihydrate (ultrapure, 98.5–101%) was obtained from Honeywell (Charlotte, North Carolina, United States); and hydrochloric acid (37%, p.a.) were bought from Merck (Darmstadt, Germany). Ultra-pure water was generated via Purelab Flex 3 (ELGA LabWater, Veolia Water Technologies Deutschland GmbH, Celle, Germany).

### 2.2. Raw materials and preparation of albedo and flavedo fractions

Sample preparation was performed according to Krygier et al. (44) with slight modifications. About 4.5 kg oranges (*Citrus sinensis*, Valencia from Uruguay) were purchased in October 2020 from a local supermarket (Rewe, Hannover, Germany). The quality of these oranges was classified as good (class 1) according to 543/2011/EU (45). The average size of the oranges was 62–66 mm in diameter (cal. 9–11) according to (EU) No 543/2011. Orange peels were separated into a flavedo and albedo fraction using vegetable peeler. Thus, 208 g fresh weight (FW) flavedo and 453 g FW albedo were obtained. The water content for the flavedo and albedo was 74 and 66%, respectively, determined by freeze-drying (Christ alpha 2–4 LSCbasic, Martin Christ, Osterode am Harz, Germany), resulting in a dry matter content of 54.8 g dry weight (DW) flavedo and 153.8 g DW albedo. Albedo and flavedo were ground in a grinder for 15 s on the highest intensity (Blendtec Classic 575, Bad Homburg, Germany).

### 2.3. Extraction methods

**Figure 1:**
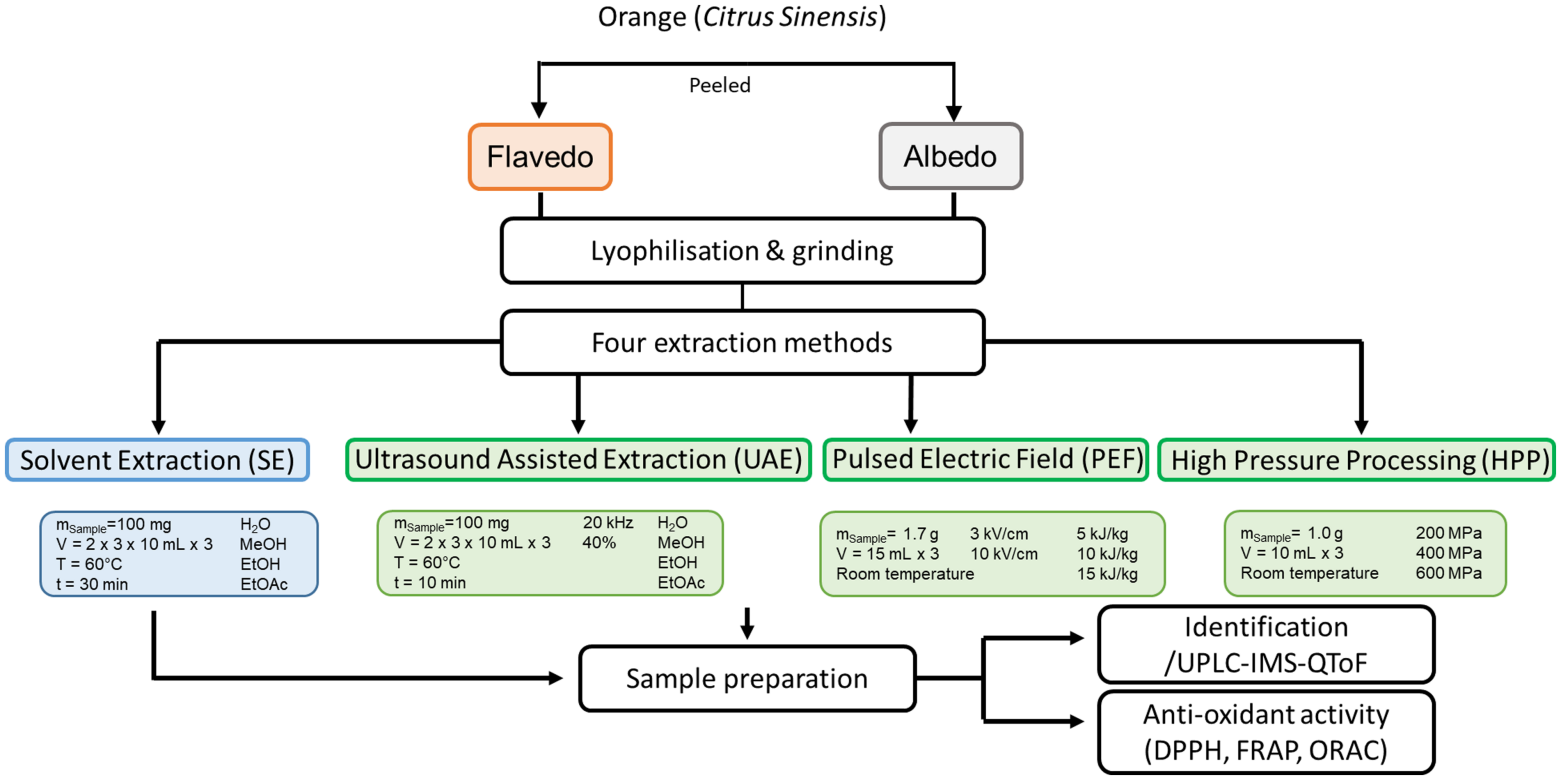
Overview of sample preparation and chemical profiling in addition to antioxidant capacity of different orange peel compartment.

To avoid large amounts of solvents each 100 mg of the sample was used for the solvent extractions and ultrasonic assisted extraction. For HHP and PEF, the amount of sample used (Figure 1) will in any case depend on previous practical experience with the respective systems, as these are industrial- or pilot-scale instruments (44, 46). Four different extraction approaches were used (Table 1). In Figure 1 a schematic diagram summarizes sample preparations.

**Table 1:** Codes for flavedo and albedo samples used for investigation.

Treatment		Flavedo	Albedo
SE (solvent extraction)	Water	SEFH	SEAH
	Methanol	SEFM	SEAM
	Ethanol	SEFE	SEAE
	Ethyl acetate	SEFA	SEAA
UAE (ultrasonic extraction)	Water	UFH	UAH
	Methanol	UFM	UAM
	Ethanol	UFE	UAE
	Ethyl acetate	UFA	UAA
PEF (pulsed electric field)	5 kJ/kg/3 kV	FP3-5	AP3-5
	10 kJ/kg/3 kV	FP3-10	AP3-10
	15 kJ/kg/3 kV	FP3-15	AP3-15
	5 kJ/kg/10 kV	FP10-5	AP10-5
	10 kJ/kg/10 kV	FP10-10	AP10-10
	15 kJ/kg/10 kV	FP10-15	AP10-15
HHP (high hydrostatic pressure)	200 MPa	FH-2	AH-2
	400 MPa	FH-4	AH-4
	600 MPa	FH-6	AH-6

#### 2.3.1. Solvent extraction

About 10 mL solvent (ultra-pure water, methanol, ethanol, and ethyl acetate) was added to 100 mg freeze-dried orange peel and extracted in a shaking water bath for 30 min at 60°C. The samples were centrifuged at 4,500 rpm for 10 min, the supernatant was collected, and 10 mL of fresh solvent was added to the samples. The extraction was repeated three times, the supernatants were combined and the solvent was removed by an evaporator. The crude extract was taken up in 5 mL of methanol. The extraction procedure was performed in triplicate.

#### 2.3.2. Ultrasound-assisted extraction

About 100 mg of flavedo and albedo were dissolved in 10 mL solvent and placed in a water bath at 60°C. Ultra-pure water, methanol, ethanol and ethyl acetate were used as solvents. The cone tip (MS 73) of the ultrasonic homogenizer (SONOPULS HD 2200.2, Bandelin electronic GmbH, Berlin, Germany) irradiated the solution non-pulsed with a nominal power of 40% for 10 min. After the sonication, the samples were centrifuged at 4,500 rpm for 10 min, the supernatant was collected, and 10 mL of fresh solvent was added to the sample. The extraction was repeated three times, the extracts were combined, and the solvent was removed using a rotary evaporator. The crude extract was resolved in 5 mL of methanol. The extraction was performed in triplicate.

#### 2.3.3. Pulsed electric fields assisted extraction

About 1.7 g of the dried and ground flavedo or albedo samples was transferred into the teflon-lined measuring cell and filled up with 15 mL tap water watering the entire measuring cell including the planar electrodes. The distance between the electrodes was 2 cm. A total of two field strengths were selected. In the first experiment, a field strength of 3 kV/cm was applied and in the second experiment 10 kV/cm pulse length was 80 μs and the number of pulses were selected to result in the specific energy input of 5, 10, or 15 kJ/kg. The PEF treatment was carried out at ambient room temperature on a 10 kW batch system (PEF Advantage P10 10 kW, Elea technology, Quakenbrück, Germany). The extraction was performed in triplicate. The water was removed by means of freeze-drying. Solutions with a concentration of 5 mg/mL were prepared from the freeze-dried extract.

#### 2.3.4. High hydrostatic pressure assisted extraction

About 1 g of the freeze-dried peel fraction was sealed with 10 mL ultra-pure water in a polyethylene/polyamide bag with a wall thickness of 90 μm. The bags were placed in the industrial-scale HHP equipment (Wave 6000/55, Hiperbaric S.A., Burgos, Spain) with water as a pressure transmitting medium. The treatment was performed for 10 min at 200, 400, and 600 MPa at ambient temperature in triplicate. Afterwards, water was removed by freeze-drying. Solutions with a concentration of 5 mg/mL were prepared from this extract.

### 2.4. UPLC-IMS-MS analysis of flavonoids

All extracts were analyzed on a Kinetex^®^ C18 (2.1 × 100 mm i.d., 1.7 μm, Phenomenex, Aschaffenburg, Germany) column using an Acquity UPLC-System. The mobile phase consisted of A: 0.05% formic acid in water and B: 0.05% formic acid in acetonitrile. At the beginning of the gradient, 0% B was started at a flow rate of 0.55 mL/min and linearly increased to 100% within 22–25 min. Finally, column was flushed with 100% B for 3 min. Thereafter, initial conditions were established within 3 min (47). The column temperature was kept constant at 50°C. The injection volume was 0.65 μL. Detection was performed using a VION-IMS-QToF (Waters™, Eschborn, Germany) mass spectrometer equipped with traveling wave ion mobility. Electrospray ionization was performed in positive ionization mode, the cone voltage was set at 40 V, and the capillary voltage was set to 2.2 kV. *N*_2_ was used as desolvation gas at a temperature of 550°C and a flow rate of 800 L/h. Data were acquired for a mass range of *m/z* 50–1,600 in sensitivity-mode at a rate of 0.4 scans per second. In MS^E^ mode, data were acquired using two channels: at low collision energy with 6 eV and with a high collision energy ramped from 20 to 50 eV for mass range of *m*/z 60–1,500. The service sample kit from Waters was used before each analysis according to manufacturer’s recommendations. At *m/z* 556, the resolution was determined to nearly 40,000 Full width at half maximum (FWHM). Every 5 min lockmass correction was performed automatically through the reference sprayer, a solution of leucine enkephaline (54 nmol/L) in acetonitrile: water (1:1; v:v) + 0.1% formic acid with a flow rate of 10 μL/min.

### 2.5. Determination of the antioxidant activity using spectrophotometric assays

#### 2.5.1. DPPH assay

The procedure was carried out in accordance to Molyneux (48). Flavedo extracts were diluted 20-fold, except for the ethyl acetate extracts that were diluted 10-fold. Albedo extracts were diluted 10-fold, while ethyl acetate extracts were undiluted. In each case, 100 μL of sample (dissolved in ethanol) mixed with 100 μL of a 10 mmol/L 2,2-diphenyl-1-picrylhydrazyl solution was added to a 96-well plate, incubated in the dark for 30 min, and examined spectroscopically at a wavelength of 515 nm using the plate reader TECAN infinite M200 (Männedorf, Switzerland). Trolox standard concentrations of 5, 10, 20, 30, 40, and 50 μmol/L were used for calibration (linearity equation *y* = 0.0052× − 0.011 with *R*^2^ = 0.9988). The results were expressed as trolox equivalents in mg/100 g dry weight extract.

#### 2.5.2. FRAP assay

A 20 mM ferric chloride hexahydrate solution was prepared with sodium acetate buffer (300 mM, pH 3.6). 2,4,6-Tri-(2-pyridyl)*-s-* triazine (TPTZ) was first mixed with 200 μL of 1 M hydrochloric acid and diluted with buffer to obtain a 10 mM solution. The FRAP reagent was prepared by adding 10 mL of acetate buffer to 1 mL each of ferric chloride hexahydrate and TPTZ. Standards were prepared at concentrations of 5, 20, 40, 60, 80, and 100 μM using the 10 mM Trolox stock solution (linearity equation *y* = 5.7656× + 0.0229 with *R*^2^ = 0.9987). All flavedo extracts were diluted 20-fold, but the ethyl acetate extract diluted 10-fold. For albedo, all extracts except the ethyl acetate extract were diluted 10-fold. Ethyl acetate albedo extract was used undiluted. In each case, 200 μL of FRAP reagent was mixed with 50 μL of sample, calibration and blank (dissolved in ethanol). The absorbance was measured at 593 nm in a plate reader. The results were expressed as Trolox equivalents in mg/100 g dry weight extract (49).

#### 2.5.3. ORAC assay

A 150 mM solution of 2, 2′-azobis-(2-methyl-propionamidine) dihydrochloride (AAPH) was prepared in phosphate buffer (75 mM, pH = 7.4) and stored on ice in the dark until use. Starting with a 100 mM sodium fluorescin stock solution, a 55 nM solution was prepared with the phosphate buffer. All extract samples were diluted 250-fold. A volume of 250 μL of the 55 nM sodium fluorescin was added to a 96 well plate and 25 μL of sample (dissolved in ethanol) was added. Then, 25 μL of the APPH solution was also added and incubated at 37°C for 10 min. Subsequently, the absorbance was measured at 520 nm after prior excitation at 485 nm. The measurement was carried out until a quenching of the fluorescence signal was observed. The total measurement time was 60 min and a measurement was performed every 5 min (50). Trolox was used for calibration [1 mM stock solution and standards with a concentration of 1, 2.5, 5, 10, 20, 25, and 50 μM (linearity equation *y* = 0.0044× + 0.186 with *R*^2^ = 0.9952)].

### 2.6. Statistical analysis

All samples were prepared and analyzed as biological triplicates. The results are presented as mean ± standard deviation. Statistical analysis was performed using SPSS software (version 26.0, SPSS Inc. Chicago, IL, Unite States). Semi-quantification was utilized according to the integrated peak areas of each metabolite. For multivariate statistical analysis, Progenesis QI and EZ Info 3.0.3 (Waters^™^, Eschborn, Germany) were used for principal component analysis (PCA) and partial least square discriminant analysis, whereas orthogonal projection to latent structures (OPLS-DA) was performed using SIMCA (Umetrics, Umeå, Sweden). Heatmaps were plotted with Knime 4.5.2 (KNIME AG) and MetaboAnalyst 5.0 (Xia Lab @ McGill, Quebec, Canada).

## 3. Results and discussion

### 3.1. Influence of the extraction technique and MS^E^ characterization of metabolites

All samples were analysed via UPLC and ESI-IMS-QToF-MS^E^. The method used allowed the matching of compounds by retention time, mass and impact cross-section based on the Waters database (UNIFI). Identification of flavonoids, especially polymethoxy flavonoids, was based on primary fragment patterns and compared with corresponding literature data (5, 51, 52), Progenesis MetaScope theoretical fragmentation patterns together with an *in silico* SciFinder database of polymethoxy flavonoids, and the Symrise database which was created via authentic compounds.

**Figure 2:**
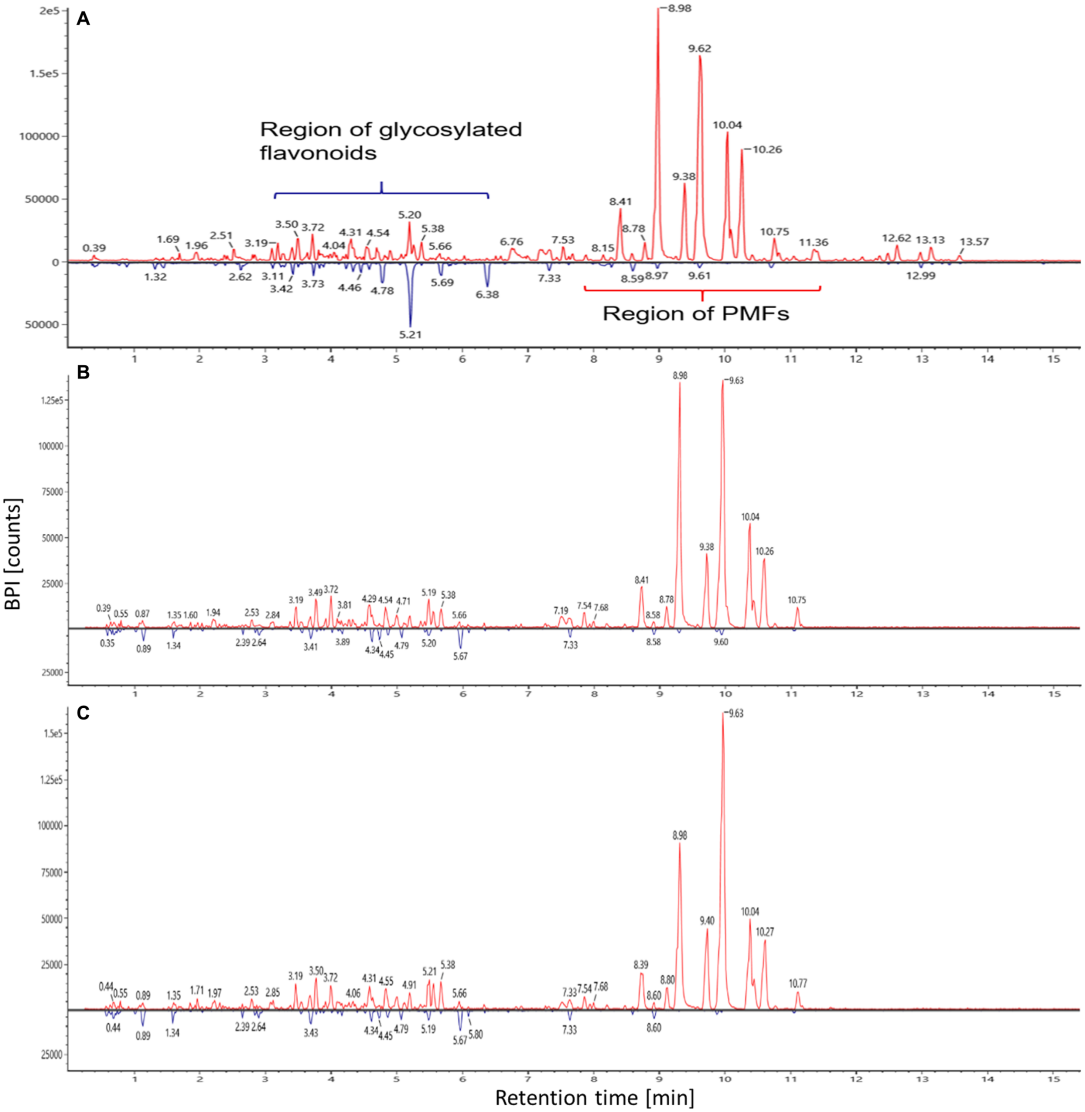
Overlay of base peak intensity (BPI) chromatograms of flavedo (in red) and albedo (in blue) samples extracted by **(A)** ethyl acetate via ultrasonic method, **(B)** HHP (400 MPa), **(C)** PEF (15 kJ/kg/3 kV).

The differences between flavedo and albedo are noticeable chromatographically. Although the main components in the flavedo were eluted in the retention range between 8 and 12 min, the majority of the components in the albedo were eluted in the time range between 3 and 7 min (Figure 2; Supplementary Figure S1). Based on the different elution time windows, it can be concluded that the majority of the compounds in the albedo are more polar than the compounds in the flavedo. In addition, the chemical profiles of flavedo and albedo were greatly different as depicted in Table 2. Considering the qualitative composition of the extracts, they had slight difference in the number of compounds. The flavedo extracts of the polar solvents showed the same spectrum of compounds (Supplementary Figure S1), the only exception being monohydroxy-tetrmethoxyflavone (peak 55), which could not be extracted by water or even via PEF and HHP. Instead, this compound was detected in the ethyl acetate extract, suggestive to its nonpolar nature. In contrast, monohydroxy-hexamethoxyflavone (peak 57) could not be detected in the ethyl acetate samples extracted by conventional and ultrasonic approaches, suggestive to its polar nature. In the albedo extracts, a flavanone glycoside (peak 15) could not be detected in ethyl acetate samples. Nevertheless, the trend can be observed that decreasing polarity of the solvent will slightly decrease the number of such polar compounds, i.e., peaks 15 and 57. This finding could be explained by the principle of “like dissolves like” consistent with reported data (79).

**Table 2:** Identified metabolites with retention time and mass characters on positive ionization of flavedo and albedo extracts resulted from four different approaches [conventional, ultrasonic, HHP (High Hydrostatic Pressure), and Pulsed Electric Field (PEF)].

No.	RT (min)	CCS (Å^2^)	Neutral mass	Formula	Metabolite	Class	Mass error (ppm)	Fragments	Flavedo	Albedo	Reference
1	0.46	258.96	738.2416	C_34_H_42_O_18_	Acacetin (di-deoxyhexosyl)-hexoside	Monomethoxy flavone glycoside	6.1	593, 447, 285	+	−	(53)
2	0.76	168.42	288.0843	C_12_H_16_O_8_	Phlorin	Phenolic glycoside	−0.7	127	+	+	(54)
3	2.64	187.26	364.1132	C_9_H_10_O_4_	Dihydrocaffeic acid dimer	Phenolic acid	−7.2	183	−	+	(55)
4	2.91	222.66	554.2929	C_32_H_42_O_8_	Khayasin	Limonoid	8.9	161	+	+	(56)
5	3.19	238.44	610.1532	C_27_H_30_O_16_	Rutin	Flavone glycoside	−0.3	465, 303	+	−	(57)
6	3.50	235.67	594.1583	C_27_H_30_O_15_	Vicenin II	Flavone glycoside	−0.3	505, 475, 415, 355	+	+	(5)
7	3.63	255.92	742.2316	C_33_H_42_O_19_	Naringin hexoside	Flavanone glycoside	−0.6	435, 273	−	+	(58)
8	3.70	201.45	446.1238	C_22_H_22_O_10_	Dihydroxy-methoxyisoflavone hexoside	Isoflavone glycoside	5.7	432, 285	+	−	(59)
9	3.73	246.84	624.1689	C_28_H_32_O_16_	Di-*C*-hexosyldiosmetin	Monomethoxy flavone glycoside	−0.2	535, 505, 385	+	−	(5)
10	3.95	197.95	448.0997	C_21_H_20_O_11_	Orientin	Flavone	−0.6	329	+	−	(60)
11	4.26	242.33	610.1892	C_28_H_34_O_15_	Neohesperidin	Monomethoxy flavanone glycoside	−0.9	345, 327, 303	−	+	(61)
12	4.31	225.22	565.155	C_26_H_29_O_14_	Pelargonidin-*O*-sambubioside	Anthocyanidin	−1.3	433, 271	+	−	(62)
13	4.34	245.87	650.2571	C_32_H_42_O_14_	Limonin hexoside	Limonoid	−0.6	−	−	+	(5)
14	4.37	132.13	194.0576	C_10_H_10_O_4_	Isoferulic acid	Phenolic acid	−1.5	−	+	+	(63)
15	4.39	224.41	580.179	C_27_H_32_O_14_	Naringin	Flavanone glycoside	−0.3	273	−	+	(5)
16	4.55	238.78	594.1583	C_27_H_30_O_15_	Kaempferol -neohesperidoside	Flavone glycoside	−0.3	449, 287	+	−	(64)
17	4.62	202.93	462.116	C_22_H_22_O_11_	*C*-Hexosyldiosmetin	Monomethoxy flavone glycoside	−0.5	448, 343	+	−	(65)
18	4.72	224.57	595.1655	C_27_H_31_O_15_	Peonidin *O*-sambubioside	Anthocyanidin	−1.3	463, 301	+	−	(66)
19	4.79	160.39	272.0682	C_15_H_12_O_5_	Naringenin	Flavanone	−0.9	153	−	+	(5)
20	5.02	202.67	472.2095	C_26_H_32_O_8_	Deacetylnomilin	Limonoid	−0.5	−	−	+	(5)
21	5.06	236.92	578.1632	C_27_H_30_O_14_	Rhoifolin	Flavone glycoside	−0.6	433, 271	+	+	(67)
22	5.12	253.83	694.2832	C_34_H_46_O_15_	Nomilin hexoside	Limonoid	−0.7	533	−	+	(5)
23	5.14	198.95	390.1337	C_20_H_22_O_8_	Hydroxy-pentamethoxy-flavanone (Norcitromitin)	Polymethoxy flavanone	5.6	−	−	+	
24	5.16	184.53	346.1074	C_18_H_18_O_7_	Dihydroxy-trimethoxyflavanone	Polymethoxy flavanone	6.4	332, 317	−	+	
25	5.19	226.61	633.1788	C_30_H_33_O_15_	Pyranocyanin A	Flavonoid glycoside	−5	488, 326	+	+	(68)
26	5.21	203.3	448.1366	C_22_H_24_O_10_	Sakuranin	Flavanone glycoside	−0.6	287	−	+	(69)
27	5.23	244.22	610.1895	C_28_H_34_O_15_	Hesperidin	Monomethoxy flavanone glycoside	−0.5	465, 303	+	+	(5)
28	5.27	244.13	608.174	C_28_H_32_O_15_	Diosmin	Monomethoxy flavone glycoside	−0.2	463, 301	+	−	(70)
29	5.29	169.05	302.0789	C_16_H_14_O_6_	Methoxynaringenin	Monomethoxy flavanone glycoside	−0.4	183, 121	+	+	(69)
30	5.38	244.27	608.174	C_28_H_32_O_15_	Neodiosmin	Monomethoxy flavone glycoside	−0.2	463, 286	+	−	(70)
31	5.40	211.07	462.116	C_22_H_22_O_11_	*O*-Hexosyldiosmetin	Monomethoxy flavone glycoside	−0.5	448, 301	+	−	(71)
32	5.67	212.49	514.2201	C_28_H_34_O_9_	Nomilin	Limonoid	−0.4	419	−	+	(5)
33	5.79	163.68	272.0682	C_15_H_12_O_5_	Chalconaringenin	Chalcone	−0.9	154, 148	−	+	(63)
34	5.80	259.43	712.2937	C_34_H_48_O_16_	Nomilinic acid -*O*-hexoside	Limonoid	−0.8	−	−	+	(69)
35	6.12	198.27	338.1506	C_21_H_22_O_4_	Bergamottin	Coumarin	−3.5	−	+	+	(57)
36	6.31	266.07	872.2576	C_38_H_48_O_23_	Kaempferol (di-deoxy-hexosyl)-pentosyl-hexoside	Flavone glycoside	−1.2	741, 595, 449, 287	+	−	
37	6.34	244.57	594.1945	C_28_H_34_O_14_	Poncirin*	Monomethoxy flavanone glycoside	−0.5	449, 287	−	+	(70)
38	6.39	164.72	286.0839	C_16_H_14_O_5_	Sakuranetin	Monomethoxy flavanone	−0.6	151	−	+	(5)
39	7.00	268.87	886.2734	C_39_H_50_O_23_	Kaempferol -isorhamninoside-rhamnoside	Flavone glycoside	−1	741, 595, 433	+	−	
40	7.19	171.38	302.0787	C_16_H_14_O_6_	Homoeriodictyol chalcone	Chalcone	−1.1	178, 154	+	−	(72)
41	7.45	318.5	1140.294	C_53_H_56_O_28_	Kaempferol *O*-sinapoyl-caffeoyl-sophoroside -*O*-hexoside	Flavone glycoside	−1.9	772	+	−	(73)
42	7.54	179.7	358.1051	C_19_H_18_O_7_	Desmethyltangeretin	Polymethoxy flavone	−0.3	328, 297, 133	+	−	(51)
43	7.73	318.5	1140.294	C_53_H_56_O_28_	Kaempferol *O*-sinapoyl-caffeoyl-sophoroside -*O*-hexoside isomer	Flavone glycoside	−1.9	772	+	−	(73)
44	8.41	187	372.121	C_20_H_20_O_7_	Auranetin	Polymethoxy flavone	0.2	358, 343, 211, 108	+	−	(57)
45	8.59	202.72	470.1937	C_26_H_30_O_8_	Limonin	Limonoid	−0.6	−	+	+	(5)
46	8.88	177.1	344.0889	C_18_H_16_O_7_	Xanthomicrol	Polymethoxy flavone	−2	330	+	−	(5)
47	8.97	186.05	372.1207	C_20_H_20_O_7_	Sinensetin	Polymethoxy flavone	−0.7	358, 211, 138	+	+	(57)
48	9.15	195.78	430.1262	C_22_H_22_O_9_	Hexamethoxy-homoflavone	Polymethoxy homoflavone	−0.4	416, 401	+	−	(74)
49	9.40	193.93	402.1315	C_21_H_22_O_8_	Hexamethylquercetagetin	Polymethoxy flavone	0.1	388, 372, 357, 341, 211	+	−	(75)
50	9.62	193.27	402.1313	C_21_H_22_O_8_	Nobiletin	Polymethoxy flavone	−0.6	388, 372, 241	+	+	(5)
51	9.65	175.15	342.1102	C_19_H_18_O_6_	Demethoxytangeretin	Polymethoxy flavone	−0.5	328, 281, 211	+	+	(76)
52	9.69	189.1	388.1156	C_20_H_20_O_8_	Demethylnobiletin	Polymethoxy flavone	−0.5	358, 330	+	−	(5)
53	9.97	193.13	430.1262	C_22_H_22_O_9_	Hexamethoxy-homoflavone isomer	Polymethoxy homoflavone	−0.4	416, 401	+	−	(74)
54	10.04	198.38	432.1422	C_22_H_24_O_9_	Heptamethoxyflavone	Polymethoxy flavone	0.4	418, 402, 387, 371	+	+	(5)
55	10.10	182.27	358.1051	C_19_H_18_O_7_	5-*O* Methylmikanin	Polymethoxy flavone	−0.3	328, 297, 149	+	−	(77)
56	10.26	184.4	372.121	C_20_H_20_O_7_	Tangeretin	Polymethoxy flavone	0.2	358, 343, 325, 297, 241	+	+	(5)
57	11.27	194.66	418.1259	C_21_H_22_O_9_	Natsudaidain	Polymethoxy flavone	−1.1	389	+	−	(78)

*Indicated authentic samples used for identification.

Distinguishment of compositional isomers is possible by means of IMS. This permits the use of the collisional cross section (CCS) as an additional identification qualifier and shorter analytical gradients. The resulted CCS data were quite reproducible during co-elution as well as when a complex matrix like orange peel was present demonstrating the reliability of the generated CCS data and their application for compound identification. Comparisons of numerous PMF isomers in the flavedo were carried out using triplicate CCS measurements (Supplementary Figure S2) to reveal the recognized correlation between CCS and *m/z.* The monohydroxy pentamethoxyflavones, having six distinct isomers, made up the largest group of isomers examined. Numerous isomers with distinct CCS could be characterized. However, isomers with the same CCS, were eluted at various periods using the adopted separation technique (Supplementary Figure S3) indicating the value of utilizing IMS coupled to tandem mass spectrometry in identification of observed isomer peaks. Small adjustments to the structural configuration can, nevertheless, frequently have a huge effect on CCS values. For instance, the dihydroxy-tetramethoxyflavones and fully methoxylated pentamethoxyflavones only differ by 2 Da, yet the dihydroxy-tetramethoxyflavones have an average 183.85 Å^2^ CCS with 4.07 Å^2^ less than the pentamethoxyflavones (average CCS of 187.92 Å^2^). This is likely due to the effects of the hydroxyl group’s positional variance and isobaric structural alterations on CCS values. Flavanones revealed larger CCS value than flavones exemplified in dihydroxy-trimethoxyflavanone and norcitromitin compared to xanthomicrol and demethylnobiletin, respectively. It is probably caused by the flavone C-ring’s planar orientation, as a result of the SP2 hybridization of the C2 atom connected to the double bond. The restricting conjugated bonds between C2 and C3 with the attached B-ring allow the C ring to be in-plane with both the A and B rings, so lowering its total spatial geometry. Contrarily, a flavanone’s orientation, which has an equatorial connection of the B ring to the C2 and no restraint conjugated bonds, permits the B ring to move freely and rotate, increasing the average spatial conformation (61).

The *Citrus* species exhibits almost exclusively the flavonoid subgroup of polymethoxyflavones, these represent interesting target compounds due to their pharmacological activity (5). The fully methoxylated flavones could be detected as molecular ion peaks in the low energy spectrum without further fragments. Both the molecular ion and the fragments are detectable in the high-energy spectrum. The sensitivity is increased and the noise is decreased due to the alignment of the low and high-energy spectra, ruling out interfering signals to display just analyte-specific signals.

The parent ion exhibit fragment masses due to methyl losses [*M*− *n* × CH_3_ +H]^+^ depending on the number of methoxy units. Tangeretin (peak number 56) was considered as a reference substance to identify the expected fragment patterns in the low-energy and high-energy spectra. In the low-energy spectrum, the molecular ion was detected at *m/z* 373.12835 [M+H]^+^ without any fragments, so it can be assumed that no fragments are expected in the low-energy. The high-energy spectrum showed the diversity of fragment formation, including signals for the characteristic fragments of methyl cleavage at *m/z* 358 [M+H-CH_3_]^+^, *m/z* 343 [M+H-2CH_3_]^+^, water cleavage at *m/z* 325 [M+H-2CH_3_-H_2_O]^+^ and CO cleavage at *m/z* 297 [M+H-2CH_3_-H_2_O-CO]^+^. Retro-Diels-Alder fragmentation processes coupled to characteristic fragments, causing cleavage in the C-ring, can be used to figure out the number and type of substituents in the A- and B-rings. In the case of tangeretin, the most obvious signal was detected at *m/z* 241 [^1,3^A+H]^+^, suggestive to completely methoxylated A-ring (Supplementary Figure S2).

Two tangeretin isomers had same molecular formula (C_20_H_20_O_7_) peaks 44, 47, albeit different fragmentation patterns were detected. Both peaks showed fragment ion at *m/z* 211 [^1,3^A+H]^+^ corresponding to tri-methoxylated A-ring. However, peak 44 produced extra fragment at *m/z* 108 suggestive to mono-methoxylated B-ring. Thus, peaks 44 and 47 were identified as auranetin and sinensetin, respectively. The order of the three isomers based on CCS values was auranetin (187 Å^2^) > sinensetin (186.05 Å^2^) > tangeretin (184.4 Å^2^) indicating that methoxyl group at position 3 had a large impact on spatial size of the molecule. Sinensetin and tangeretin were detected in flavedo and albedo samples, although auranetin was present exclusively in the flavedo part. Another study on *Citrus reticulata* confirmed the finding that sinensetin had larger CCS value in respect to tangeretin indicating a more compact structure of the latter (80).

Peaks 19 and 33 had the same parent ion at *m/z* 273.0754 [M+H]^+^. Peak 19 yielded fragment ion at *m/z* 153 owing to [^1,3^A+H]^+^ cleavage. However, peak 33 had characteristic fragment ions at *m/z* 154, 148 corresponding to cleavage around the carbonyl group. Therefore, peaks 19 and 33 were annotated as naringenin and chalconaringenin, respectively. Chalconaringenin is a typical chalconoid that has been discovered for the first time in lemon peel and has the ability to spontaneously cyclize to naringenin (81). The CCS of chalconaringenin (163.68 Å^2^) was higher than naringenin (160.39 Å^2^) attributed to the increased size of analyzed ion via the opening of the central ring in naringenin. Both naringenin and chalconaringenin were detected only in albedo part. Likewise, peaks 29 and 40 were identified as methoxynaringenin and homoeriodictyol chalcone, respectively. Flavedo and albedo samples encompassed methoxynaringenin, albeit homoeriodictyol chalcone was present exclusively in flavedo part. Noteworthy, chalchones were eluted after their corresponding flavonoid, albeit the former had higher CCS value attributed to larger spatial size. Peaks 28 and 30 had the same molecular formula C_28_H_32_O_15_ and were identified exclusively in flavedo part as diosmin and neodiosmin, respectively. Although the retention behavior of the two isomers is quite close, they can be distinguished from one another based on their MS spectra. Diosmin was the sole component to elute with *m/z* 301 fragment owing to aglycone ion, while neodiosmin lacks *m/z* 301 fragment ion (70). Diosmin revealed lower CCS value (ΔCCS, 0.14 Å^2^) compared to neodiosmin. Remarkably, two isomers, peaks 11 and 27, shared the same molecular formula (C_28_H_34_O_15_) and ion fragment at *m/z* 303 due to hesperetin aglycone. However, extra fragment ions at *m/z* 345, and 327 were exclusively observed in peak 11. Thus, peaks 11 and 27 were identified as neohesperidin and hesperidin, respectively (61). Hesperidin exhibited higher CCS value (ΔCCS, 1.89 Å^2^) compared to neohesperidin in accordance with a previous study (82). Hesperidin was detected in both flavedo and albedo samples while, neohesperidin was present only in the albedo.

Another two flavonoids (peaks 17, 31) were detected in flavedo samples only with molecular ion at *m/z* 463.1232 [M+H]^+^. Peak 17 exhibited fragment ion at *m/z* 343 [M+H-120]^+^ attributed to *C*-hexoside cleavage. In contrast, peak 31 yielded fragment ion at *m/z* 301 [M+H-162]^+^ due to *O*-hexoside loss. Thus, peaks 17 and 31 were annotated as *C*-hexosyldiosmetin and *O* hexosyldiosmetin, respectively. *C*-Hexosyldiosmetin was eluted first with lower CCS value (ΔCCS, 8.14 Å^2^) compared to *O*-hexosyldiosmetin indicating maybe the effect of sugar linkage.

Peaks 49 and 50 had same molecular weight C_21_H_22_O_8_, yet different fragmentation pattern was observed. Peak 49 formed fragment ion at *m/z* 211 [^1,3^A+H]^+^ corresponding to tri-methoxylated A ring, while peak 50 produced fragment ion at 241 [^1,3^A+H]^+^, suggestive to completely methoxylated A ring. Therefore, peaks 49 and 50 were identified as hexamethylquercetagetin and nobiletin, respectively. Nobiletin (193.27 Å^2^) was detected with less CCS value in both flavedo and albedo parts, however hexamethylquercetagetin (193.93 Å^2^) was found exclusively in flavedo part. Likewise, peaks 42 and 55 with molecular formula C_19_H_18_O_7_ formed fragment ions at *m/z* 133 and 149, respectively due to [^1,3^B+H]^+^ cleavage. Accordingly, peaks 42 and 55 were identified as desmethyltangeretin (gardenin B) and 5-*O* methylmikanin (3-hydroxy-4′,5,6,7-tetramethoxyflavone), respectively. Gardenin B and 5-*O* methylmikanin were present only in flavedo. 5-*O* Methylmikanin exhibited higher CCS value (ΔCCS, 2.57 Å^2^) indicating that hydroxy group at position 3 increased the spatial size of the molecule. The occupation of position 3 in flavonoids by hydroxyl or methoxyl group increased CCS value compared to isomers with unoccupied carbon 3. This is evidenced by CCS values of auranetin, hexamethylquercetagetin and 5-*O* methylmikanin in respect to other isomers. Peaks 6 and 16 had the same parent ion at *m/z* 595.1655 [M+H]^+^. Peak 6 revealed fragment ions at *m/z* 505 [M+H-90]^+^, 475 [M+H-120]^+^, 415 [M+H-90-90]^+^, and 355 [M+H-120-120]^+^ indicating the presence of di-*C*-hexosyl groups. Peak 6 was annotated as apigenin-di-*C*-hexoside [vicenin II, (5)] and found in flavedo and albedo parts. Conversely, peak 16 formed fragment ions at *m/z* 449 [M+H-146]^+^, and 287 [M+H-146-162]^+^, suggestive to the presence of *O*-deoxyhexosyl-hexosyl group. Therefore, peak 10 was identified as kaempferol -neohesperidoside present only in flavedo part. Vicenin II had lower CCS value (ΔCCS, 3.11 Å^2^) compared to kaempferol-neohesperidoside.

### 3.2. Antioxidant activity

Table 3 encompassed total antioxidant activity assessed via FRAP, ORAC, and DPPH assays as mean values of three replicates (±SD). The DPPH value was significantly (*p* < 0.001) higher in flavedo (nearly double) in respect to albedo, except for albedo samples extracted by ethyl acetate via conventional and ultrasonic methods where higher values were obtained compared to flavedo. Moreover, the FRAP assay confirmed the greater antioxidant activity of flavedo compared to albedo, except for albedo samples extracted by ethyl acetate via conventional method. The ORAC test revealed that albedo had stronger antioxidant activity than flavedo extracted by conventional and ultrasonic methods, with a statistically significant difference (*p* < 0.05), except for flavedo samples extracted by water. In details, flavedo samples extracted by water via conventional method had higher ORAC values in respect to albedo, while flavedo and albedo samples extracted by water via ultrasonic method revealed non-significant difference. Furthermore, ORAC values of flavedo extracted by PEF and HHP were higher than albedo samples, except for flavedo and albedo samples extracted by HHP at 600 MPa which had non-significant difference. The enrichment of flavedo with polymethoxy flavonoids in respect to albedo partially explains the higher antioxidant activity as determined by three different methods. In all three methods, flavedo extracted by PEF and HHP had greater antioxidant activity than albedo. However, different ORAC results were observed in samples extracted by conventional and ultrasonic methods, most likely because of the limited precision of ORAC assay. In the same context, naringin and hesperidin had higher contents after extraction with ultrasonic method than conventional one resulting in enhanced antioxidant property of the extract (83). Additionally, another former study revealed that low power UAE was a suitable method for extracting highly valuable bioactive compositions from mandarin peel (84).

**Table 3:** Antioxidant activity determined by three various methods, FRAP, ORAC, and DPPH of flavedo and albedo samples extracted by four different extraction approaches.

Treatment		DPPH/mg TE/100 g		FRAP/mg TE/100 g		ORAC/mg TE/100 g	
		Flavedo	Albedo	Flavedo	Albedo	Flavedo	Albedo
SE	Water	1,451 ± 34^a^	630 ± 172^a^	1,480 ± 41^a^	467 ± 56^a^	28,113 ± 148^a^	15,170 ± 190^a^
	Methanol	1,194 ± 39^b^	393 ± 34^b^	1846 ± 228^b^	886 ± 35^b^	20,782 ± 178^b^	40,899 ± 133^b^
	Ethanol	767 ± 45^c^	394 ± 101^b^	1,425 ± 237^a^	734 ± 44^c^	15,721 ± 553^c^	42,679 ± 174^c^
	Ethyl acetate	52 ± 27^d^	1,040 ± 194^c^	1,424 ± 6,010^a^	2,376 ± 609^d^	14,334 ± 1944^d^	146,096 ± 445^d^
UAE	Water	1,373 ± 46^a^	629 ± 53^a^	1,342 ± 163^a^	522 ± 54^a^	26,607 ± 147^a^	26,563 ± 285^a^
	Methanol	1,240 ± 58^b^	432 ± 71^b^	2,105 ± 121^b^	788 ± 144^b^	26,402 ± 157^a^	38,306 ± 167^b^
	Ethanol	823 ± 17^c^	398 ± 15^b^	1,419 ± 203^c^	707 ± 59^b^	24,212 ± 117^b^	43,884 ± 101^c^
	Ethyl acetate	88 ± 13^d^	783 ± 83^c^	1882 ± 100^d^	1,409 ± 182^c^	38,669 ± 152^c^	140,086 ± 284^d^
PEF	5 kJ/kg/3 kV	2,528 ± 90^a^	924 ± 20^a^	4,469 ± 196^a^	1,119 ± 21^a^	23,771 ± 219^a^	12,401 ± 176^a^
	10 kJ/kg/3 kV	2,446 ± 146^a^	934 ± 67^a^	4,621 ± 86^a^	1,129 ± 78^a^	31,264 ± 246^b^	11,144 ± 193^b^
	15 kJ/kg/3 kV	2,617 ± 60^a^	859 ± 13^b^	4,608 ± 165^a^	1,072 ± 14^b^	30,314 ± 201^b^	6,981 ± 145^c^
	5 kJ/kg/10 kV	2,450 ± 87^a^	838 ± 50^b^	4,275 ± 160^a^	1,025 ± 38^b^	29,624 ± 352^b^	7,798 ± 165^d^
	10 kJ/kg/10 kV	2,594 ± 11^a^	922 ± 75^a^	4,519 ± 111^a^	1,059 ± 36^b^	30,991 ± 318^b^	13,137 ± 59^e^
	15 kJ/kg/10 kV	2,529 ± 69^a^	937 ± 51^a^	4,457 ± 76^a^	1,100 ± 86^a^	29,920 ± 172^b^	14,030 ± 123^f^
HHP	200 MPa	2,588 ± 28^a^	809 ± 13^a^	4,346 ± 39^a^	911 ± 23^a^	26,530 ± 262^a^	10,097 ± 128^a^
	400 MPa	2,668 ± 41^b^	730 ± 51^b^	4,462 ± 16^b^	789 ± 24^b^	24,740 ± 138^b^	4,591 ± 151^b^
	600 MPa	1,373 ± 36^c^	629 ± 53^c^	1,342 ± 163^c^	522 ± 54^c^	26,607 ± 147^a^	26,563 ± 285^c^

The values are expressed as mean ± SD with color gradient increasing from green-yellow-red. Different letters (a, b, c, d) within the same panel indicated significant difference between samples. SE, solvent extraction; UAE, ultrasonic extraction; PEF, pulsed electric field; HHP, high hydrostatic pressure; TE, trolox equivalents in mg/100 g dry weight extract.

### 3.3. Multivariate analysis of the data

**Figure 3:**
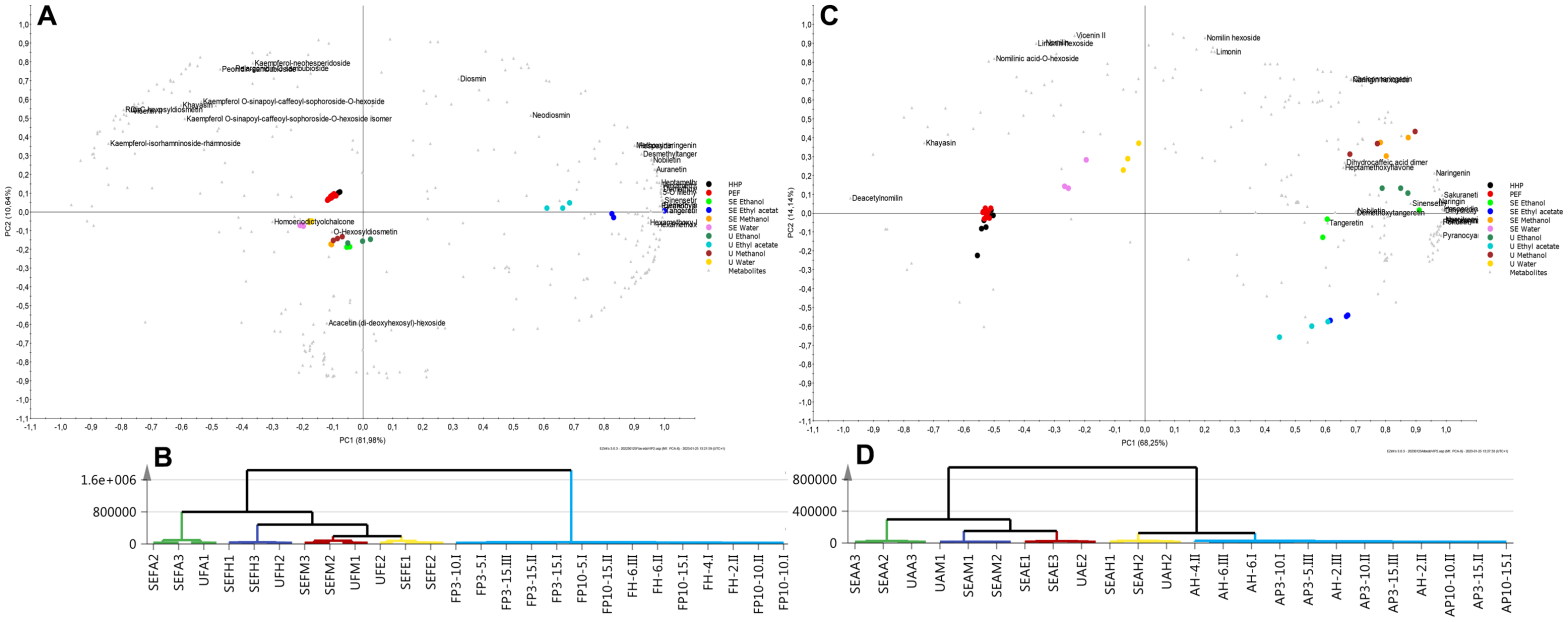
Supervised multivariate data analysis based on the ESI-IMS-QToF-MSE spectra. For flavedo samples: **(A)** biplot, **(B)** HCA dendrogram. For albedo samples: **(C)** biplot, **(D)** HCA dendrogram. Sample codes: U = Ultrasonic, SE = Solvent extraction, P = PEF, H = HHP, A = Albedo, F = Flavedo, H = Water, M = Methanol, E = Ethanol, A = Ethyl acetate or see Table 1.

Potential discriminatory metabolites from ESI-IMS-QToF-MS^E^ dataset can be highlighted using multivariate analysis. To analyze the data set’s intrinsic variation, PCA, an unsupervised pattern recognition technique, was used (85). In PCA score plots of albedo and flavedo parts (Figure 3) with total variance 79.8% and 90.65%, respectively described that, samples extracted by ethyl acetate, ethanol and methanol are clustered with positive PC1 values, whereas samples extracted by water, PEF and HHP were separated with negative PC1 values. Moreover, ethyl acetate samples formed the most distant cluster as confirmed by HCA (hierarchical clustering analysis) dendrogram. PLS-DA biplot graph of flavedo samples (Figure 3B) revealed that kaempferol glycosides, rutin, and orientin contributed to distinguish samples extracted by water, PEF, and HHP posing sizeable amounts of those metabolites. However, samples extracted by ethyl acetate tended to cluster on the upper right-hand quarter owing to the enrichment with large number of metabolites, i.e., auranetin, 5-*O* methylmikanin, hexamethoxy-homoflavone, and neodiosmin. On the other hand, PLS-DA biplot graph of albedo samples (Figure 3C) demonstrated that deacetylnomilin contributed to distinguish samples extracted by PEF and HHP. Albedo samples extracted by water were enriched with vicenin II, limonin hexoside and nomilinic acid -*O*-hexoside, attributed to the high polarity of these metabolites. Naringin and chalconaringenin were the most discriminatory metabolites in albedo samples extracted by ethanol, while naringenin, sakuranetin and hesperidin were the most abundant components in samples extracted by methanol.

A supervised pattern recognition algorithm (OPLS-DA) was suitable for the detection of discriminatory components in comparable metabolic profile data in order to further discover ion peaks that can discriminate between the groups (86). The OPLS-DA of flavedo extracted by ethyl acetate against all other solvents, i.e., ethanol, methanol, and water made it possible to resolve the most significant discriminatory metabolites in ethyl acetate extract (Supplementary Figure S4). The S-loading graph (Supplementary Figure S4B) showed that hexamethoxy-homoflavone and its isomer were the distinctive discriminatory metabolites in ethyl acetate extract. Another OPLS-DA (Supplementary Figure S4C) was performed to compare flavedo samples extracted by methanol via conventional and ultrasonic methods. The effect of ultrasonic approach using the same solvent was characterized by higher levels of fully methylated PMFs, i.e., heptamethoxyflavone, hexamethylquercetagetin, tangeretin, and nobiletin (Supplementary Figure S4D). However, the conventional solvent extraction had no discriminatory metabolites. Similar findings in respect to nobiletin and tangeretin were previously reported, where both metabolites extracted from red orange peel via UAE were increased by 1.5 times compared to conventional solvent extraction (87). Likewise, OPLS-DA was conducted between ultrasonic and conventional techniques using water (Supplementary Figure S5). The S-loading plot (Supplementary Figure S5B) revealed that conventional aqueous extract encompassed higher levels of partially methoxylated flavonoids, i.e., desmethyltangeretin and demethylnobiletin, while the related ultrasonic approach showed no markers. Furthermore, OPLS-DA was performed to pinpoint the impact of changing pressure on HHP extracted flavedo samples. High pressure (600 MPa) treatment did not reveal any discriminatory metabolites, yet lower pressure (200 MPa) treatment demonstrated enrichment with sinensetin (Supplementary Figure S5D). Regarding albedo samples, OPLS-DA was carried out between water and ethyl acetate to investigate the impact of solvent nature (Supplementary Figure S6). The S-loading plot (Supplementary Figure S6B) showed that glycoside metabolites, i.e., naringin hexoside, limonin hexoside, and nomilin hexoside were abundant in aqueous extracts, while pyranocyanin A was the discriminatory marker for ethyl acetate extracts. Lastly, low-energy input of PEF at 5 kJ/kg/3 kV was compared to high energy input at 15 kJ/kg/3 kV using OPLS-DA (Supplementary Figure S6C). The S-loading plot (Supplementary Figure S6D) showed that higher energy input resulted in PEF extracts enriched with limonoids, i.e., nomilin and limonin. In the same context, Luengo et al. (88) reported elevated contents of naringin and hesperidin upon orange peels extraction using PEF at 5 kV. To visualize in an intuitive way how well the relevant metabolites can distinguish between various extraction techniques heat-map was performed (Supplementary Figure S7). Each metabolite is represented by one rectangle, which is colored according to a normalized scale from −4 (low) to 4 (high). In flavedo samples, two metabolites, i.e., homoeriodictyol chalcone, and khayasin were up-regulated in conventional aqueous extracts. *O*-Hexosyldiosmetin was up-regulated in conventional methanol extracts. In contrast, methoxynaringenin was down-regulated in conventional ethyl acetate extracts. In albedo samples, two metabolites, i.e., tangeretin and heptamethoxyflavone were up-regulated in conventional ethanol extracts. Three metabolites, i.e., nomilinic acid-*O*-hexoside, limonin hexoside, and nomilin, were down-regulated in samples extracted by HHP at low pressure (200 MPa).

The results of antioxidant activity suggest that flavedo has better antioxidant activity than albedo because of the variety of chromatographic profiles and the distribution of various flavonoid chemical classes demonstrating their complex composition. Unsupervised multivariate data analysis revealed that the solvent polarity represented the discriminating factor to segregate various clusters. Supervised multivariate data analysis could explain the resulted antioxidant activity to some extent. Ethyl acetate extract of flavedo samples exhibited higher antioxidant activity due to enrichment with hexamethoxy-homoflavone compared to other solvents. However, methoxynaringenin showed strong negative correlation with ethyl acetate (Supplementary Figure S7). Fully methoxylated PMFs, i.e., heptamethoxyflavone and hexamethylquercetagetin were the main reason of higher antioxidant activity in flavedo samples obtained by ultrasonic technique using methanol compared to solvent extraction method. In contrast, partially methoxylated metabolites, i.e., desmethyltangeretin, demethylnobiletin, homoeriodictyol chalcone, and khayasin were responsible for elevated antioxidant activity in aqueous flavedo extracts obtained by conventional method. Untraditional approaches causing cell wall damage, i.e., PEF and HHP resulted in raised antioxidant activity of flavedo samples owing to enrichment with kaempferol glycosides. However, high pressure at 600 MPa may cause loss of some metabolites, i.e., sinensetin resulting in lower antioxidant activity. Thus, HHP at medium pressure (400 MPa) was the optimum condition for flavedo extraction (Figure 2B) attributed to the potential presence of higher content of sinensetin. According to Escobedo-Avellaneda et al. (89), the current utilized medium pressure of HHP was comparable to the appropriate range of 450–550 MPa previously reported. In contrast, a lower pressure at 100 MPa was reported by M’hiri et al. (90) as optimum condition to obtain the highest antioxidant activity at 11.9 μM Trolox equivalent. For best practice, extraction of flavedo using PEF at 15 kJ/kg/3 kV (Figure 2C) was the most efficient approach concerning antioxidant activity. Regarding the effect of solvent, water was the best solvent to conventionally extract flavedo due to enrichment with partially methoxylated metabolites. However, methanol was the most appropriate solvent to extract flavedo using UAE due to enrichment with fully methylated PMFs. In the same context, ethyl acetate proved to be the solvent of choice to extract albedo using conventional or UAE methods. The majority of researchers reported that methanol increased phenolic content extracted from citrus peel (91). Conversely, ethyl acetate was reported to produce the least phenolic content compared to methanol and ethanol (92). Glycoside metabolites, i.e., naringin hexoside, limonin hexoside, and nomilin hexoside were abundant in aqueous extracts of albedo, while pyranocyanin A was abundant in ethyl acetate samples and account for the higher antioxidant effect of the latter. The optimum condition for albedo extraction using PEF was 15 kJ/kg/10 kV. Therefore, higher energy input (15 kJ/kg) is desirable for extraction of both flavedo and albedo using PEF. Likewise, El Kantar et al. (93) reported an improved flavonoid content and total phenolic contents of flavedo and albedo using PEF at 10 kV/cm. HHP at low pressure (200 MPa) proved to be more efficient particularly in albedo part regarding antioxidant effects, although the loss of some limonoids suggestive to the greater impact of flavoinoids compared to other classes. For best practice, conventional extraction of albedo using ethyl acetate was the most efficient approach concerning antioxidant activity attributed to the enrichment with pyranocyanin A.

## 4. Conclusion

This study provided here impels the purpose of advancing industrial practices toward selection of the proper extraction technique by serving as a foundation for future work obtaining specific individual flavonoids particularly polymethoxy flavones (PMFs) from biomass residues. Extracts obtained via modern technologies, particularly pulsed electric field and high pressure processing, were consistently richer in antioxidants rather than conventional solvent extraction. This current research article highlighted how CCS values can be used to link metabolite structure and function, and discriminate between 21 isomers in a complex matrix, thus improving the confidence in metabolite identification important for ensuring the food authenticity and quality of highly esteemed products in order to prevent fraud. The findings of this study reveal that orange flavedo and albedo tissues can be used for medicinal and nutraceutical reasons. Furthermore, it shows differences in profile in dependence on the extraction approach used, having HHP and PEF yielding relatively similar composition, compared to other studied techniques.

Having different CCS values (ΔCCS, 2.65 Å^2^), hexamethoxy-homoflavone and its isomer present exclusively for the first time in flavedo part should be isolated and analyzed by 2D NMR spectroscopy as a future aspect to pinpoint the specific position of methoxyl groups discriminating between the two isomers. It is possible to create a database with retention data, CCS values and mass spectra using the findings from the analysis of various orange peel extracts under the same experimental conditions to confirm the presence of flavonoid metabolites in other citrus peel, or less thoroughly explored food matrices containing flavonoids. Future research should focus on how total and/or individual citrus PMFs correlate with biological engineering techniques including breeding, genetic engineering, and fermentation engineering to accomplish cross-application of production.

## Data availability statement

The raw data supporting the conclusions of this article will be made available by the authors, without undue reservation.

## Author contributions

RG, SH, and TE contributed to conception and design of the study. SA, IE, DK, ER, and A-SS performed the analysis. SA, IE, and ER performed the statistical analysis. SA wrote the first draft of the manuscript. DK wrote sections of the manuscript. All authors contributed to the article and approved the submitted version.

## Funding

This research project was partially supported by the German academic exchange program (DAAD; German Egyptian Research Short-Term Scholarship—GERSS program). The publication of this article was funded by the Open Access Fund of Leibniz Universität Hannover.

## Conflict of interest

IE, ER, and SH were employed by Symrise AG.

The remaining authors declare that the research was conducted in the absence of any commercial or financial relationships that could be construed as a potential conflict of interest.

## Publisher’s note

All claims expressed in this article are solely those of the authors and do not necessarily represent those of their affiliated organizations, or those of the publisher, the editors and the reviewers. Any product that may be evaluated in this article, or claim that may be made by its manufacturer, is not guaranteed or endorsed by the publisher.
