# Supplementary material for: Comparative flavonoid profile of orange (Citrus sinensis) flavedo and albedo extracted by conventional and emerging techniques using UPLC-IMS-MS, chemometrics and antioxidant effects
Source: Front Nutr. 2023 Jun 6;10:1158473. doi: 10.3389/fnut.2023.1158473 (PMC10279959; doi:10.3389/fnut.2023.1158473)
Supplement: Supplementary file 1 [file Data_Sheet_1.docx]

Supplementary Material

Comparative flavonoid profile of orange (*Citrus sinensis*) peel extracted by conventional and emerging techniques using UPLC-IMS-MS, chemometrics and antioxidant effects

Sherif M. Afifi^1,2^, Recep Gök^3^, Ingo Eikenberg^4^, Dennis Krygier^1^, Eric Rottmann^4^, Anne-Sophie Stübler^5,^ Kemal Aganovic^5^, Silke Hillebrand^4^, Tuba Esatbeyoglu^1^*

^1^ Institute of Food Science and Human Nutrition, Gottfried Wilhelm Leibniz Universität Hannover, 30167 Hannover, Germany [esatbeyoglu@lw.uni-hannover.de](mailto:esatbeyoglu@lw.uni-hannover.de), [d.krygier@aol.de](mailto:d.krygier@aol.de)

^2^ Pharmacognosy Department, Faculty of Pharmacy, University of Sadat City, Sadat City 32897, Egypt; [shshsh38@hotmail.com](mailto:shshsh38@hotmail.com)

^3^ Institute of Food Chemistry, Technische Universität Braunschweig, 38106 Braunschweig, Germany; [r.goek@tu-braunschweig.de](mailto:r.goek@tu-braunschweig.de)

^4^ Symrise AG, 37603 Holzminden, Germany; [silke.hillebrand@symrise.com](mailto:silke.hillebrand@symrise.com), [eric.rottmann@symrise.com](mailto:eric.rottmann@symrise.com), [ingo.eikenberg@symrise.com](mailto:ingo.eikenberg@symrise.com)

^5^ German Institute of Food Technologies (DIL e.V.), 49610 Quakenbrück, Germany [k.aganovic@dil-ev.de](mailto:k.aganovic@dil-ev.de), [annasophie.stuebler@gmail.com](mailto:annasophie.stuebler@gmail.com)

*** Correspondence:**Prof. Dr. Tuba Esatbeyoglu
esatbeyoglu@lw.uni-hannover.de

# Supplementary Figures


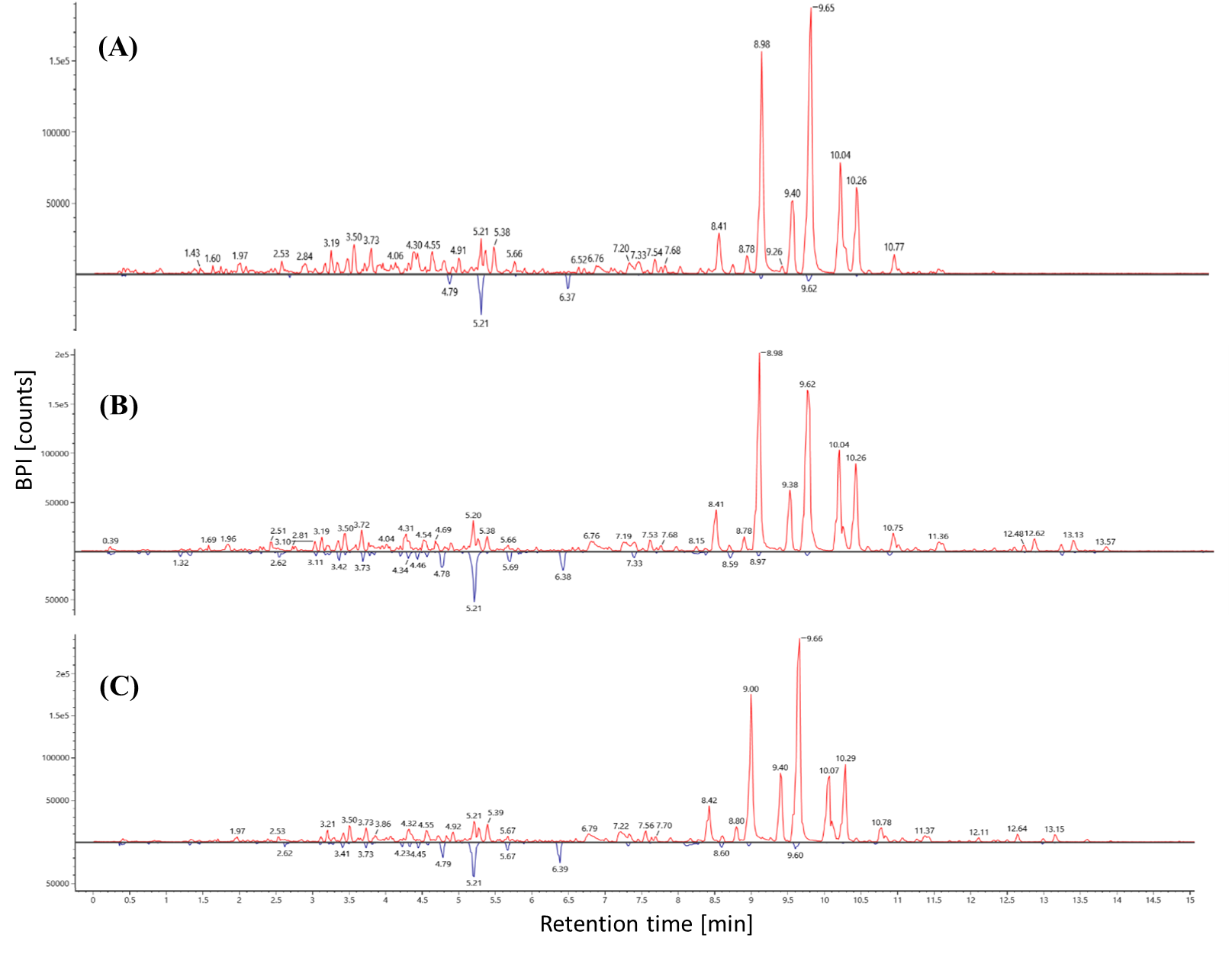


**Supplementary Figure** 1. Overlay of base peak intensity (BPI) chromatograms of flavedo (in red) and albedo (in blue) samples extracted via ultrasonic method by A: water, B: methanol, C: ethanol.


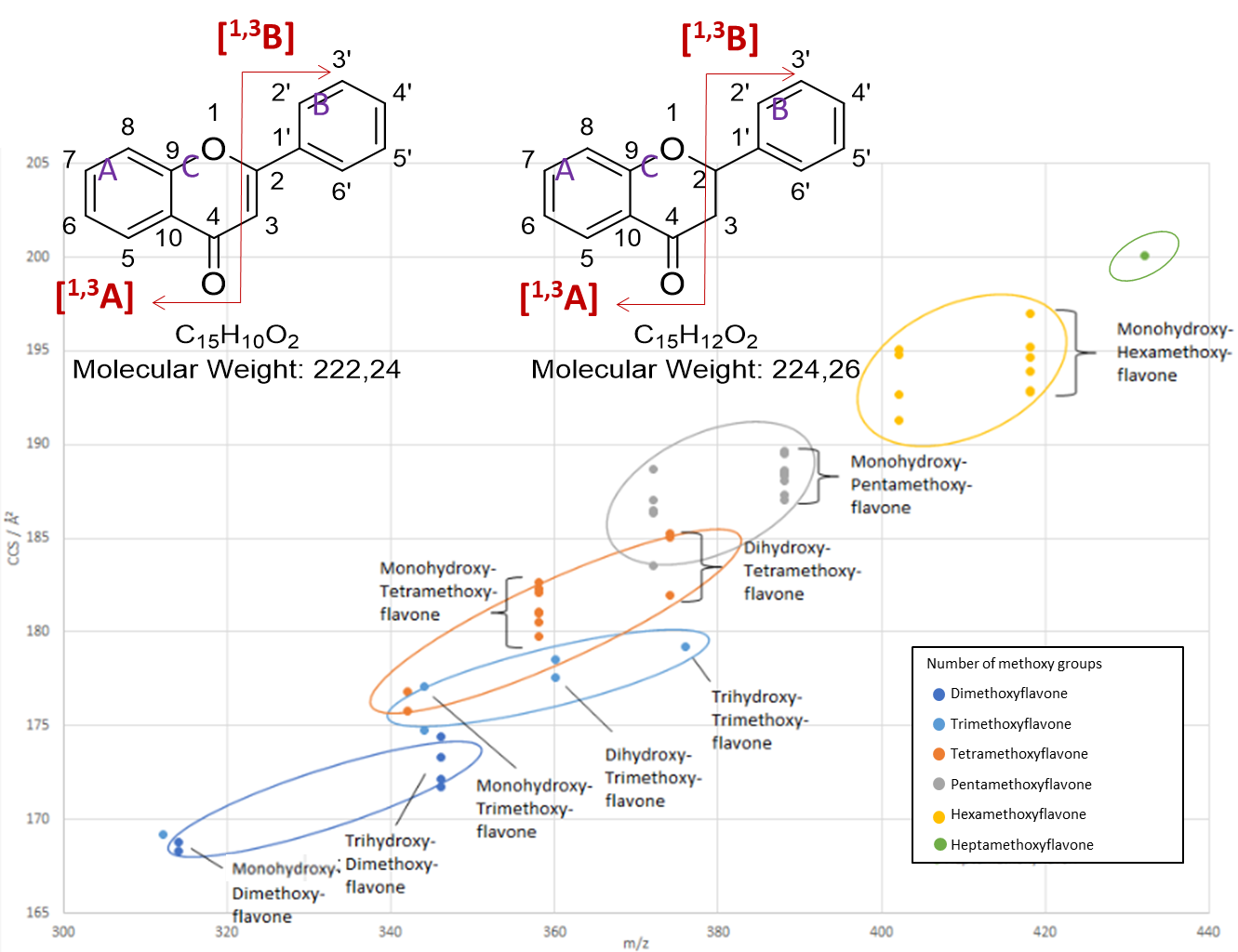


**Supplementary Figure** 2. Correlation between relative ion mobility-derived collision cross section (CCS) distribution and *m/z* of various methoxylated flavonoid isomers. In addition, basic flavone (on left) and flavanone (on right) ring configurations, with functional locations and highlighted Retro-Diels-Alder fragmentation processes.


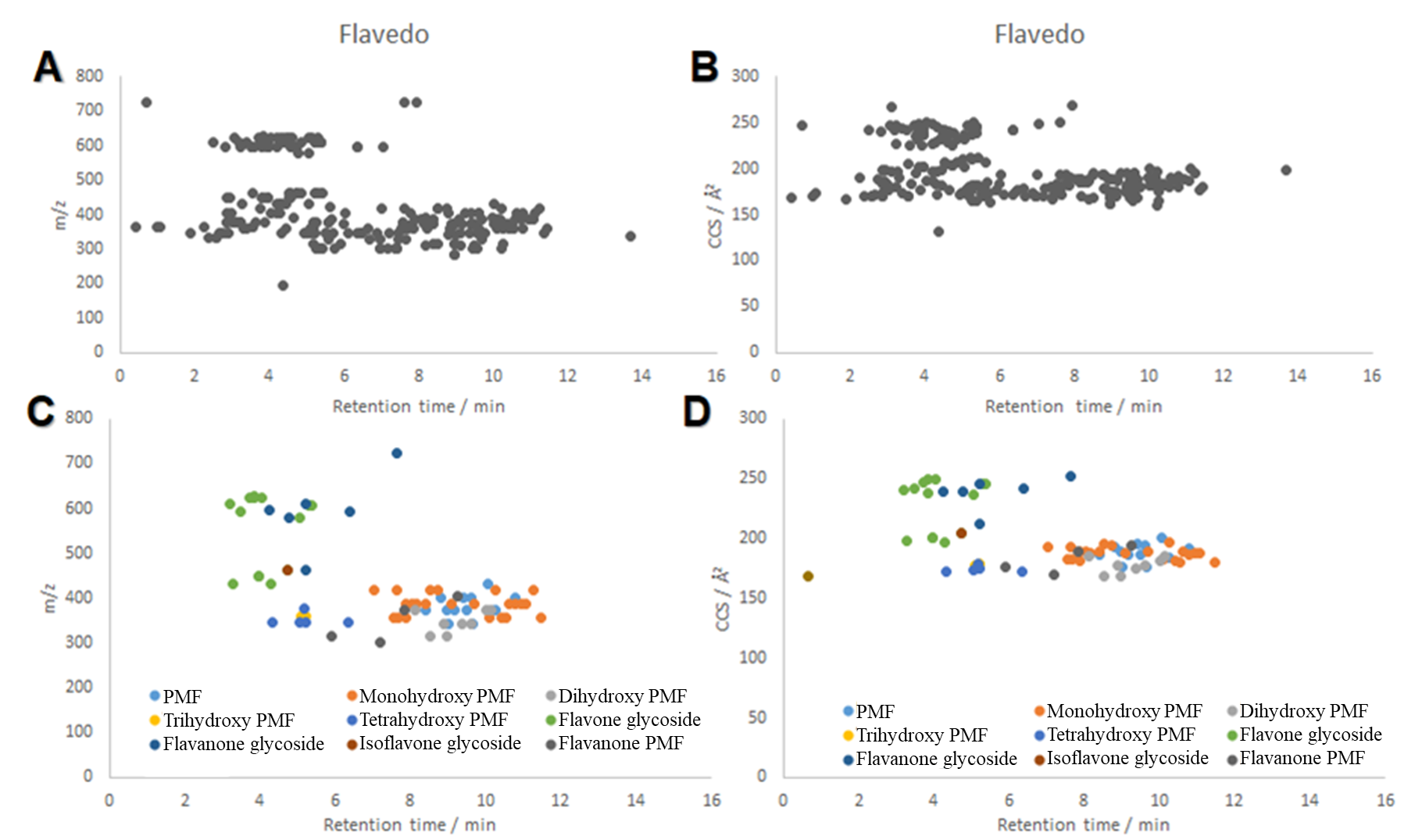


**Supplementary Figure** 3. Ion map for flavedo in terms of retention time and *m/z* (A) and retention time and CCS (B) for all flavonoid masses detected in positive mode. C and D highlight the flavonoid isomers. PMFs: polymethoxy flavones.


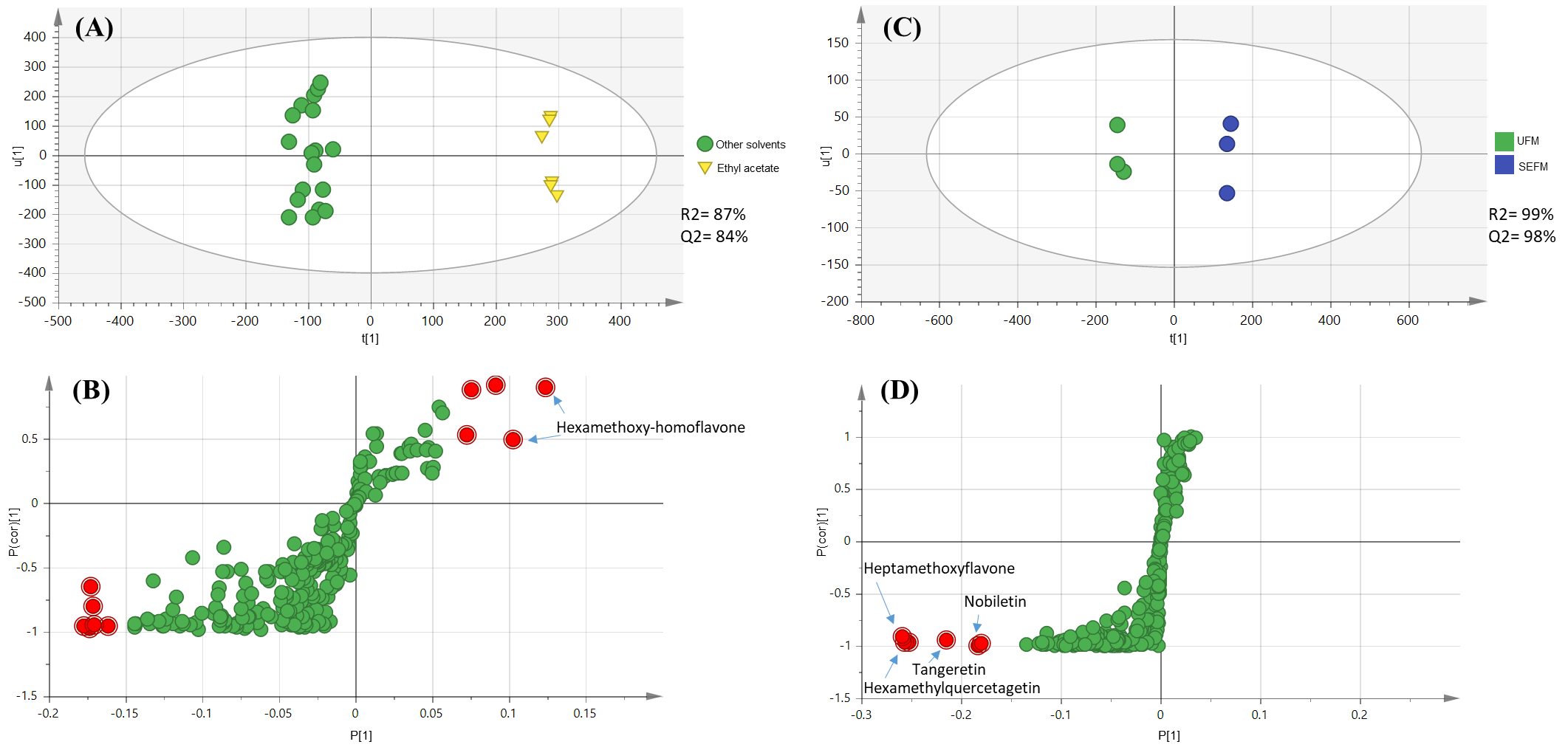


**Supplementary Figure** 4. Supervised multivariate data analysis based on the UHPLC-Q-TOF MS spectra of flavedo: OPLS-DA of ethyl acetate against all other solvents A: score plot, B: S-loading plot; and OPLS-DA of methanol extracted by ultrasonic against conventional method C: score plot, D: S-loading plot.


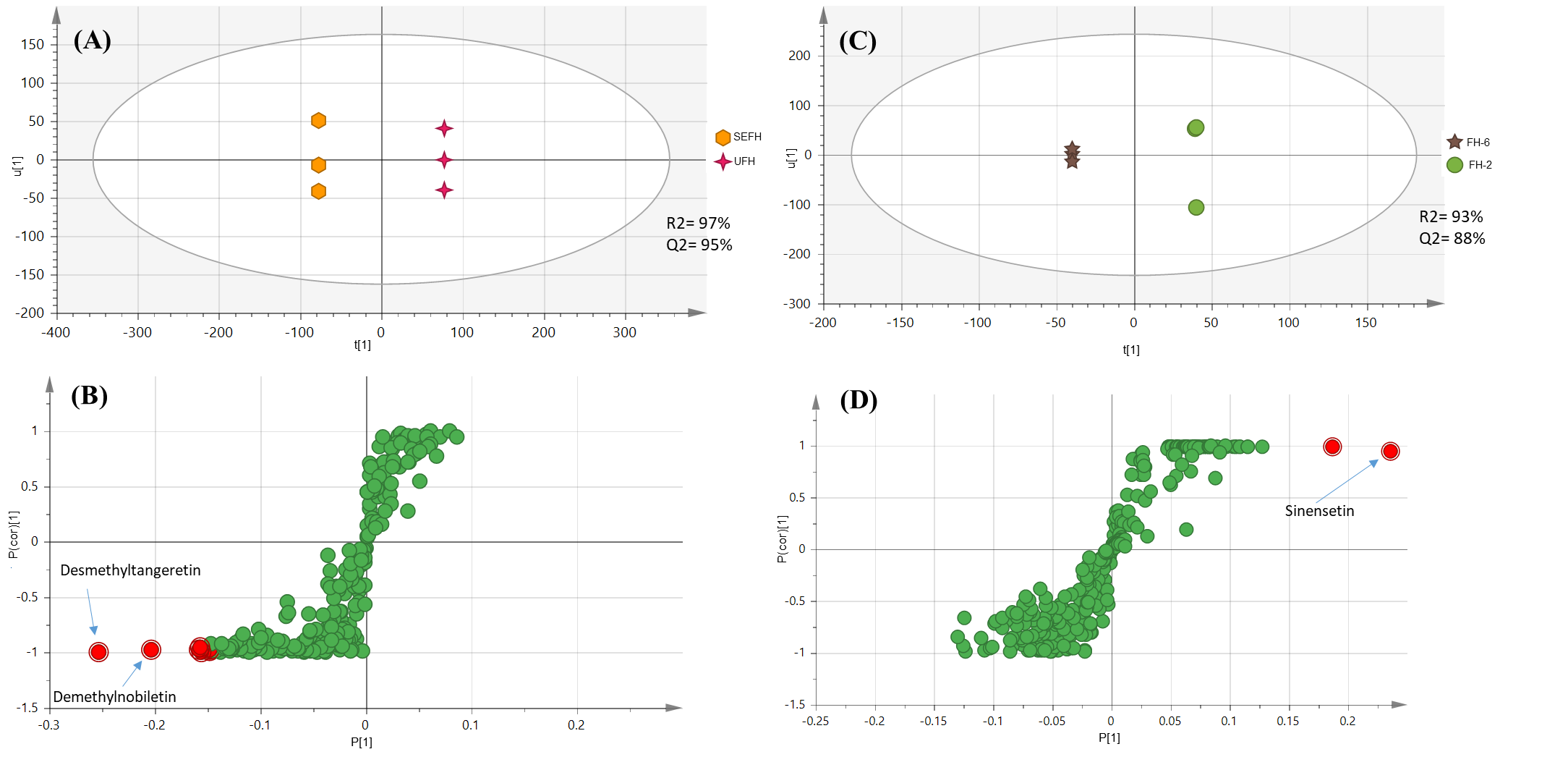


**Supplementary Figure** 5. Supervised multivariate data analysis based on the UHPLC-Q-TOF MS spectra of flavedo: OPLS-DA of aqueous samples extracted by conventional against ultrasonic method A: score plot, B: S-loading plot; and OPLS-DA of HHP samples extracted at 200 MPa against 600 MPa C: score plot, D: S-loading plot.


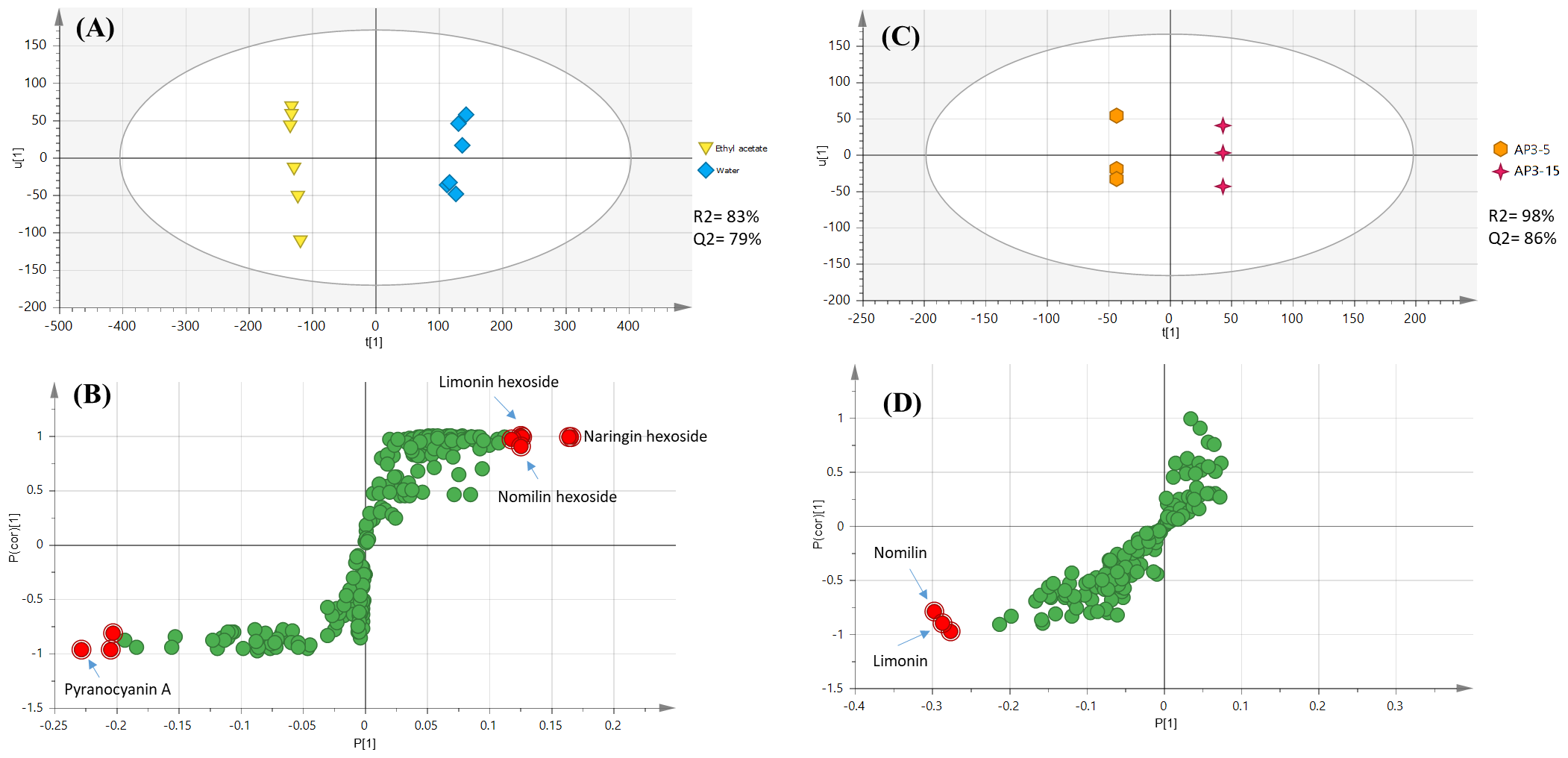


**Supplementary Figure** 6. Supervised multivariate data analysis based on the UHPLC-Q-TOF MS spectra of albedo: OPLS-DA of aqueous extracts against ethyl acetate extracts A: score plot, B: S-loading plot and OPLS-DA of PEF samples extracted at 15 kJ/kg / 3kV against 5 kJ/kg /3kV C: score plot, D: S‑loading plot.

**Supplementary Figure** 7. Heat map demonstrating potential biomarker variations in levels in both flavedo (A) and albedo (B) samples. Columns representing various samples; rows representing biomarkers. The color key displays the ratio of the biomarker content (blue: lowest; red: highest). HHP: High Hydrostatic Pressure, PEF: Pulsed Electric Field. Sample codes: U = Ultrasonic, SE = Solvent extraction, P = PEF, H = HHP, A = Albedo, F = Flavedo, H = Water, M = Methanol E =Ethanol, A = Ethyl acetate or see Table 1.





**Tangeretin**

**Heptamethoxyflavone**

**Nomilinic acid -*O*-hexoside**

**Limonin hexoside**

**Nomilin**


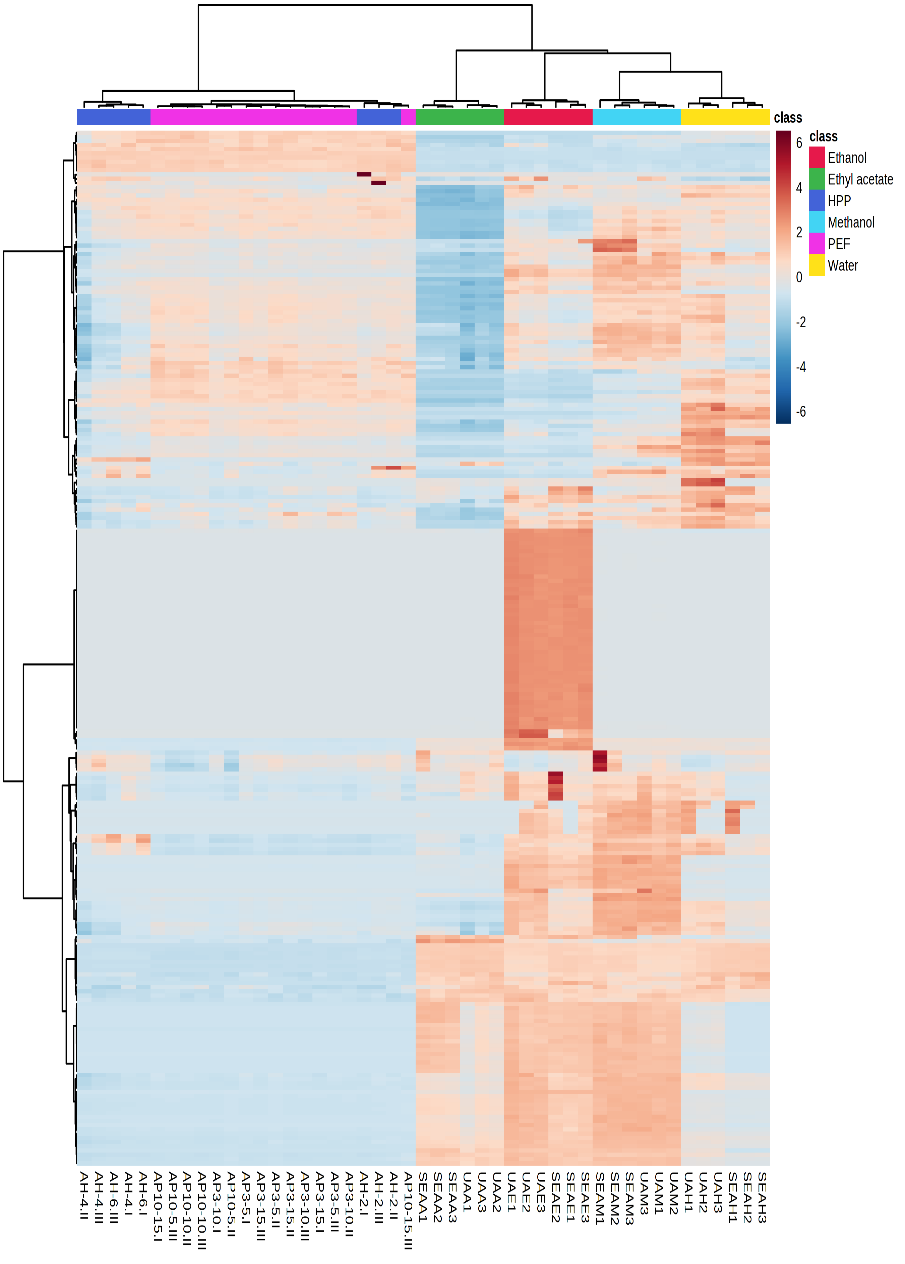


**(B)**

***O*-Hexosyldiosmetin**

**Homoeriodictyol chalcone**

**Khayasin**

**Methoxynaringenin**

**Acacetin (di-deoxyhexosyl)-hexoside**


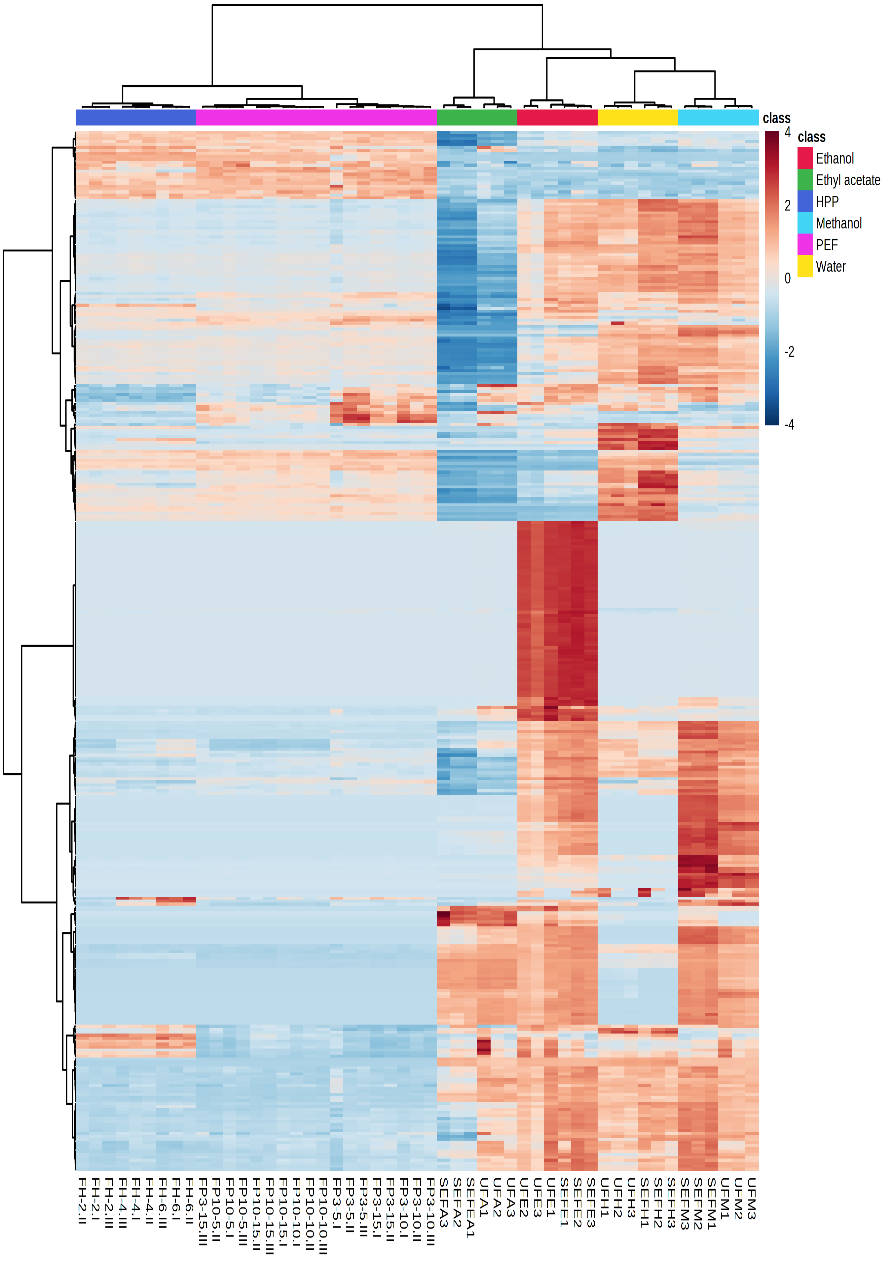


**(A)**
